# Surgical Treatment of Giant Cavernous Hemangioma Liver

**DOI:** 10.1155/1991/91486

**Published:** 1991

**Authors:** V. A. Vishnevsky, V. S. Mohan, V. S. Pomelov, F. I. Todua, E. K. Guseinov

**Affiliations:** 1 Dept. Surgical Gastroenterology Vishnevsky Institute of Surgery Academy of Medical Sciences USA; 2 Block 12/Flat 10 MIG (11) Baghlingampally Hyderabad 500 044 India; 3 Dept. Computed Tomography Vishnevsky Institute of Surgery Academy of Medical Sciences USA; 4 Dept. Interventional Radiology Vishnevsky Institute of Surgery Academy of Medical Sciences USA

## Abstract

In the past five years, 16 adults (10 females, age 25–61 years, mean 48) with giant cavernous hemangioma of the liver measuring 15–31 cm (mean-19) underwent surgery in a single Institution. Diagnosis was made with the help of multimodal investigations– ultrasound (US), computed tomography (CT), hepatic angiography, hepatic scintigraphy and fine needle biopsy. Ultrasound and CT had sensitivities of 69% and 82% respectively. Fourteen had preoperative selective hepatic artery embolization to study its effect on operative blood loss. Indication for surgery in all cases was a large abdominal mass with varying severity of pain. In addition, 5 had hemetological and/or coagulation abnormalities, hemobilia in and pyrexia in 1. Seven left lobectomies, 3 left lateral segmentectomies, 2 right lobectomies, 2 right trisegmentectomies and 4 non-anatomical resections of to 3 segments were performed. Postoperative complications developed in 25% with no operative mortality. Preoperative selective hepatic artery embolization helped to decrease the operative hemorrhage in 13 (mean blood loss– 1146 ml). In two cases severe bleeding required use of Cell-saver and massive donor blood transfusion. Our results suggest use of preoperative selective hepatic artery embolization and Cell-saver as an adjunct to the liver resection for these vascular tumors.


*HPB Surgery*, 1991. Vol. 4, pp 69–79
Reprints available directly from the publisher
Photocopying permitted by license only

© 1991 Harwood Academic Publishers GmbH
Printed in the United Kingdom

### SURGICAL TREATMENT OF GIANT CAVERNOUS HEMANGIOMA LIVER

V.A. VISHNEVSKY

*Senior Research Scholar, Dept. Surgical Gastroenterology, Vishnevsky Institute of Surgery, Academy of Medical Sciences*

V.S. MOHAN*

*Ph. D. Scholar, Dept. Surgical GE, Vishnevsky Institute of Surgery, AMS*

V.S. POMELOV

*Professor and Head, Dept. Surgical GE, Vishnevsky Institute of Surgery, AMS*

F.I. TODUA

*Professor and Head, Dept. Computed Tomography, Vishnevsky Institute of Surgery, AMS*

E.K. GUSEINOV

*Junior Research Scholar, Dept. Interventional Radiology, Vishnevsky Institute of Surgery, AMS*

*(Received 5 November 1990)*

In the past five years, 16 adults (10 females, age 25–61 years, mean 48) with giant cavernous hemangioma of the liver measuring 15–31 cm (mean-19) underwent surgery in a single Institution. Diagnosis was made with the help of multimodal investigations– ultrasound (US), computed tomography (CT), hepatic angiography, hepatic scintigraphy and fine needle biopsy. Ultrasound and CT had sensitivities of 69% and 82% respectively. Fourteen had preoperative selective hepatic artery embolization to study its effect on operative blood loss. Indication for surgery in all cases was a large abdominal mass with varying severity of pain. In addition, 5 had hemetological and/or coagulation abnormalities, hemobilia in 1 and pyrexia in 1. Seven left lobectomies, 3 left lateral segmentectomies, 2 right lobectomies, 2 right trisegmentectomies and 4 non-anatomical resections of 1 to 3 segments were performed. Postoperative complications developed in 25% with no operative mortality. Preoperative selective hepatic artery embolization helped to decrease the operative hemorrhage in 13 (mean blood loss– 1146 ml). In two cases severe bleeding required use of Cell-saver and massive donor blood transfusion. Our results suggest use of preoperative selective hepatic artery embolization and Cell-saver as an adjunct to the liver resection for these vascular tumors.

KEY WORDS: Giant cavernous hemangioma, embolization, liver resection

*Address for Correspondence: Dr. V.S. Mohan, M.S, Ph.D (Surgical GE), Block 12/Flat 10, MIG (11), Baghlingampally, Hyderabad 500 044, India

### INTRODUCTION

Cavernous hemangioma of the liver was first described by Dupuytren and Cruveilhier[1]. It is the most common benign hepatic neoplasm with autopsy incidence of 0.7–7.4%[2,3]. In the recent years, because of widespread use of noninvasive diagnostic techniques- ultrasonography (US), computed tomography (CT) and magnetic resonance imaging (MRI), they are detected more frequently[4,5]. Natural history of the disease is vague and the role of surgery is a matter of debate[6]. However, liver resection, hepatic artery ligation and in recent years hepatic artery embolization are recommended as treatment modalities in giant cavernous hemangiomas of the liver[7–10]. In this paper clinical presentation, diagnosis and surgical treatment of 16 cases of giant cavernous hemangiomas of the liver are reviewed and the effect of preoperative embolization on the intraoperative blood loss is studied.

### PATIENTS AND METHODS

Between March 1985 and March 1990, 16 patients with giant cavernous hemangiomas of the liver underwent major hepatic resections at Vishnevsky Institute of Surgery, Academy of Medical Sciences, Moscow. Our Institute is one of the major referral centres for performing hepatic surgery in USSR. Patients with giant hepatic hemangiomas measuring 15 cm or more in size were included in this study. US, CT, hepatic angiography and hepatic scintigraphy were performed in all (Table 1). Fine needle aspiration cytology was done in 11 cases to study its diagnostic accuracy. Hepatic angiography was performed in all using standard Seldinger technique via the right or left femoral artery and this procedure was followed by selective embolization of the feeding vessel(s) of the lesion in 14 cases with the use of HYDROGEL emboli. These emboli were worked out at our Institute in collaboration with Institute of Macromolecular Chemistry, Czechoslovak Academy of Sciences. In 2 cases, embolization could not be performed because of tortusity of hepatic artery at its origin. Intraoperative ultrasound was used to define the relations of the hemangioma with surrounding hepatic parenchymal structures thus enabling safe resection with ultrasound scalpel (ALOKA SUS-101). In 2 cases HAEMONETICS Cell-saver 4 was used for transfusion of the blood from the operating field. The cut surface of liver remnant was treated by various methods- pneumothermocoagulation, biological tissue glue and omentopexy. Fourteen patients were followed-up for 6 to 45 months period.

**Table 1** Details of multimodality diagnostic procedures.

| *Procedure* | *No. of Patients* |
|---|---|
| US/CT/ANGIO/SCINTI | 16+ |
| FNAC | 11 |
| LAPAROSCOPY | 4 |
| LAPAROTOMY | 1 |

US Ultrasonography
CT Computed tomography
ANGIO Hepatic angiography
FNAC Fine nedle aspiration cytology
SCINTI Hepatic scintigraphy
\+ 14 underwent selective hepatic artery embolization preoperatively.

### RESULTS

There were 10 females and 6 males with mean age of 48 years (range 25–61 yrs). All were symptomatic with duration from 6 months to 9 years (mean 3.5 years). Palpable abdominal lump and pain of varying severity or feeling of heaviness upper abdomen were recorded in all 16 cases (Table 2). Nine had upper gastrointestinal pressure symptoms– feeling of discomfort in right hypochondrium or epigastrium, especially after food intake and nausea. Weakness and weight loss were recorded in 5, hemobilia in 1 and pyrexia in 1. Blood examination revealed anemia in 3, hypofibrinogenemia in 3 and thrombocytopenia with low prothrombin time in 1. Four patients were referred to us after laparoscopic diagnosis of the lesion and one after cholecystectomy when a patient was found to have a giant hepatic hemangioma. Ultrasound examination was diagnostic in 11 with sensitivity of 69% and showed mixed echo pattern of the lesion. CT with contrast enhancement was more helpful in diagnosis with sensitivity of 82% (13 cases). The CT features were hypodense area of the lesion on plain scan and progressive dense accumulation of contrast from periphery to centre of hemangioma on IV bolous dynamic scanning (Figures 1a & b). Fine needle aspiration cytology was done under US/CT in 11 cases without any complications. Cytological examination revealed presence of endothelial cells in 7 (64%) and elements of blood only in 4. However, presence of malignant or atypical cells was excluded in all. Selective hepatic angiography showed typical cotton-wool appearance in all (Figure 2a). Following selective embolization of the feeding vessel(s) of hepatic hemangioma (Figure 2b) in total of 14 cases, exacerbation of abdominal pain occured in 8, febrile reaction in 5 and hypofibrinogenemia in 2. Patients underwent surgery 1–12 days following embolization. Hepatic scintigraphy showed nonspecific filling defect or decreased accumulation of isotope in the lesion.

**Table 2** Clinical features and hematological abnormalities.

| *Description* | *No. of patients* |
|---|---|
| CLINICAL FEATURES | |
| Abdominal lump | |
| Pain/Heaviness upper abdomen | 16 |
| Post praundial upper abdominal discomfort/Nausea | 9 |
| Weakness and weight loss | 5 |
| Hemobilia | 1 |
| Pyrexia | 1 |
| HEMATOLOGICAL ABNORMALITIES | |
| Anemia | 3 |
| Hypofibrinogenemia | 3 |
| Thrombocytopenia and low PTI | 1 |

Multiple segmental involvement of liver was recognized in all, with hemangioma affecting segments of right lobe only in 3, left lobe only in 5 and both lobes in 8 (Table 3). The size of lesion ranged from 15 to 31 cm (mean 19). Six had additional lesions measuring 2–8 cm. Presence of huge hepatomegaly with varying severity of pain/discomfort in the abdomen was the indication for surgery in all. Other

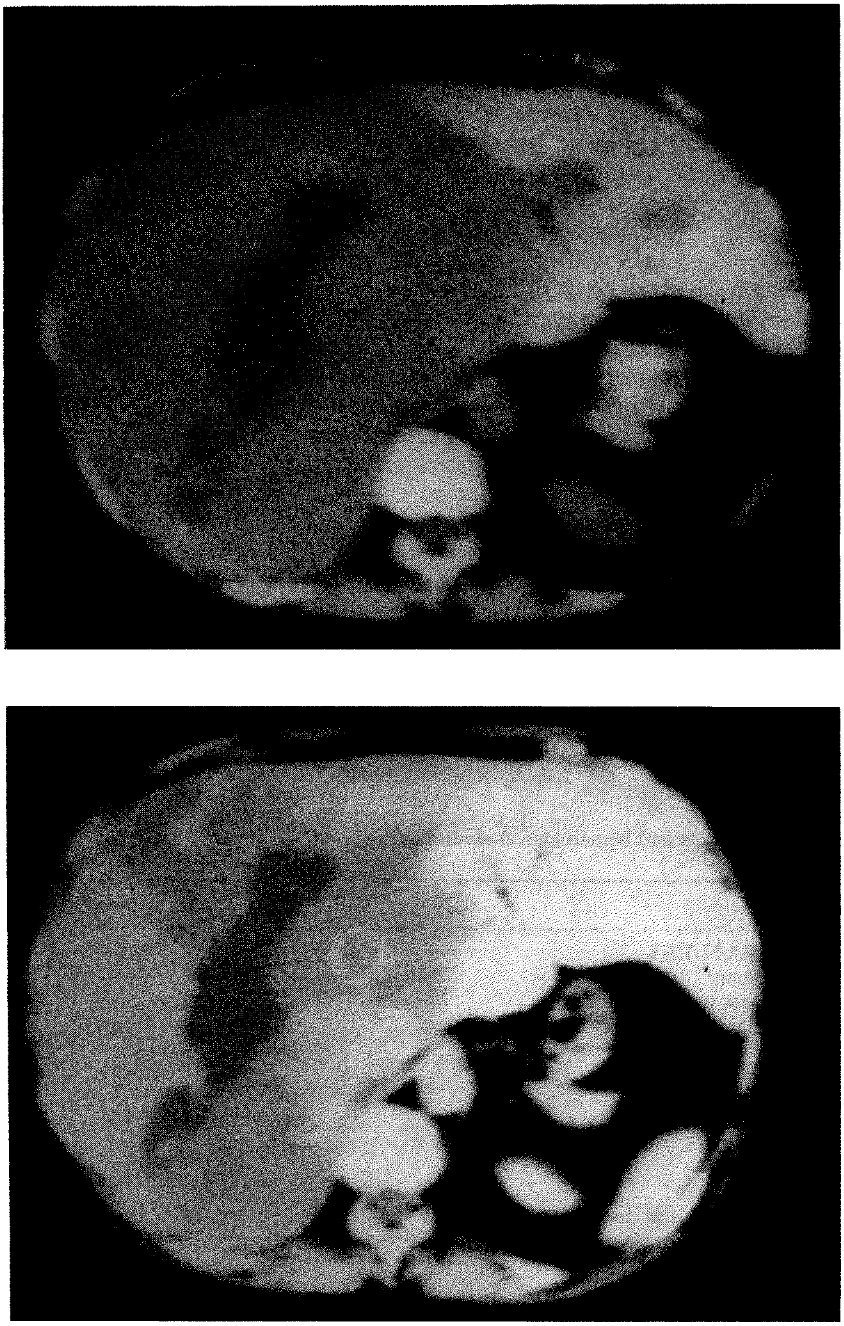

Figure 1(a). Plain CT scan (Case 15) showing huge hemangioma of right lobe liver with large central blood filled cavity. (b) Early phase of dense contrast accumulation after IV bolous injection.

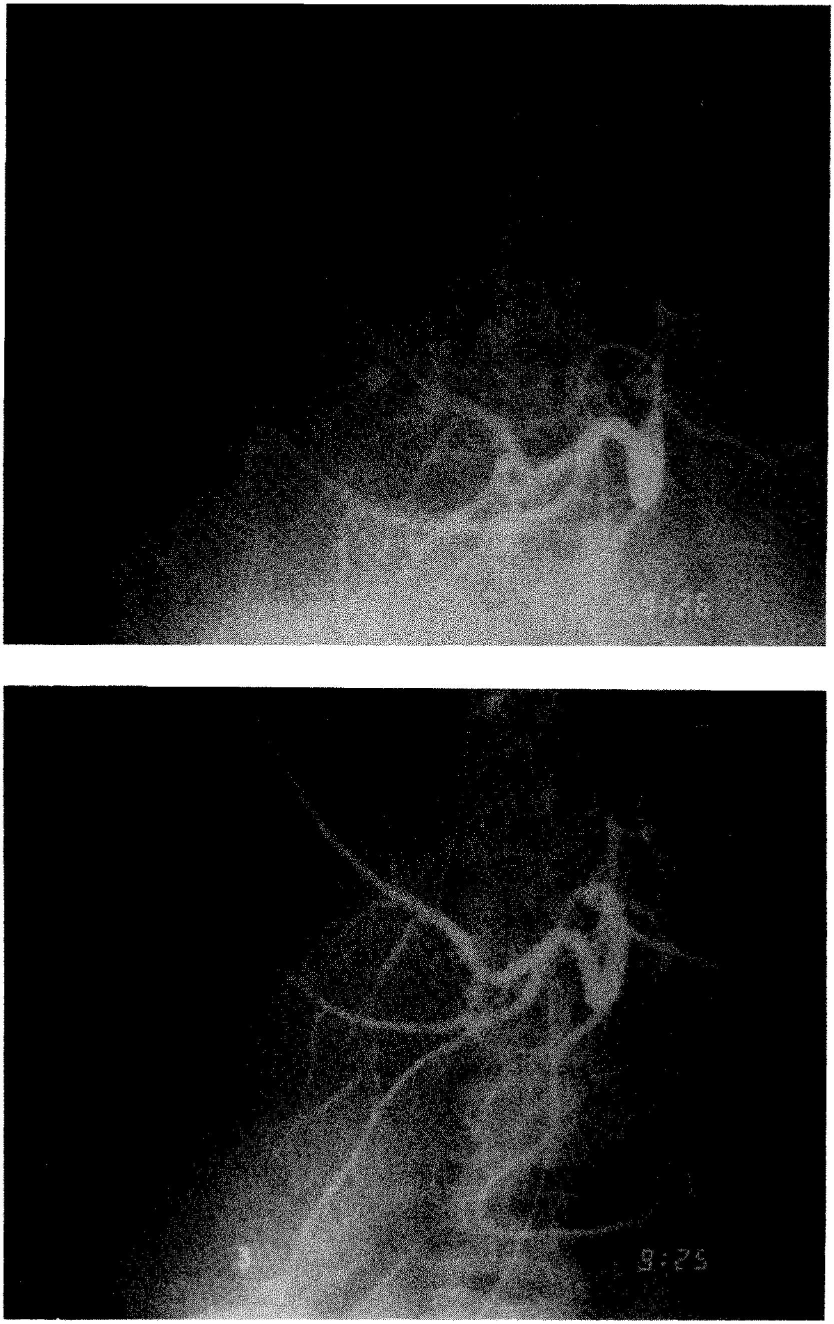

Figure 2(a). Arterial phase of hepatic angiography showing displacement of branches and diffuse pooling of the contrast (case 15). (b) Arterial phase of the same lesion following selective embolization.

indications were associated hematological and/or coagulation abnormalities in 5, rapid increase in size in 2, hemobilia in 1 and pyrexia in 1. Surgical approach was through right thoracophrenolaparotomy in 6 and bilateral subcostal in 10. Surgical procedures included (Table 3) – left lobectomies 7(43.75%), left lateral segmentectomies 3(18.75%), right lobectomies 2(12.5%), right trisegmentectomies 2(12.5%) (Figure 3a) and nonantomical resection of 2 to 3 segments in 2(12.5%). Two patients required additional resection of 1 to 2 segments and in 3, small lesions of 2–3 cm size were left unexcised. In 13 cases with embolization, operative blood loss ranged from 250 ml — 2500 ml (mean 1146) and in 2, one with and other without embolization (cases 15 and 16) the blood loss was massive– 18000 and 11000 ml respectively, requiring the use of Haemonetics Cell-saver for autotransfusion of 6000 and 4000 ml of blood respectively. The hemangiomas appeared relatively less turgid and shrinken in size following embolization. The resected specimen weighed between 460 and 3200g (mean 980) and cut section showed central cavity in four (Figure 3b). Histopathological examination confirmed the presence of cavernous

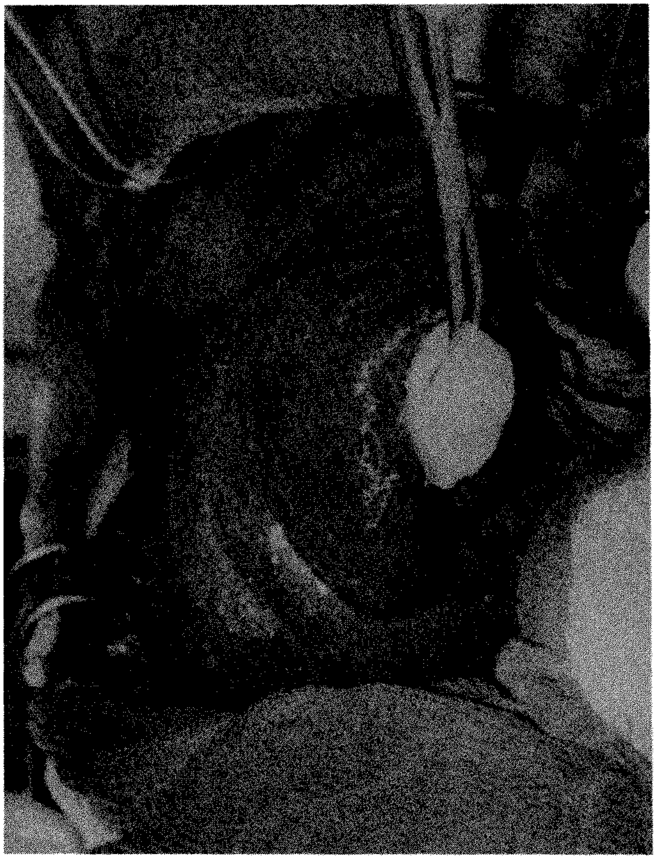

Figure 3(a). Intraoperative photograph of hemangioma (case 15). (b) Cut section showing typical shrinkage of the lesion and large central area of hyalinization and cavitation.

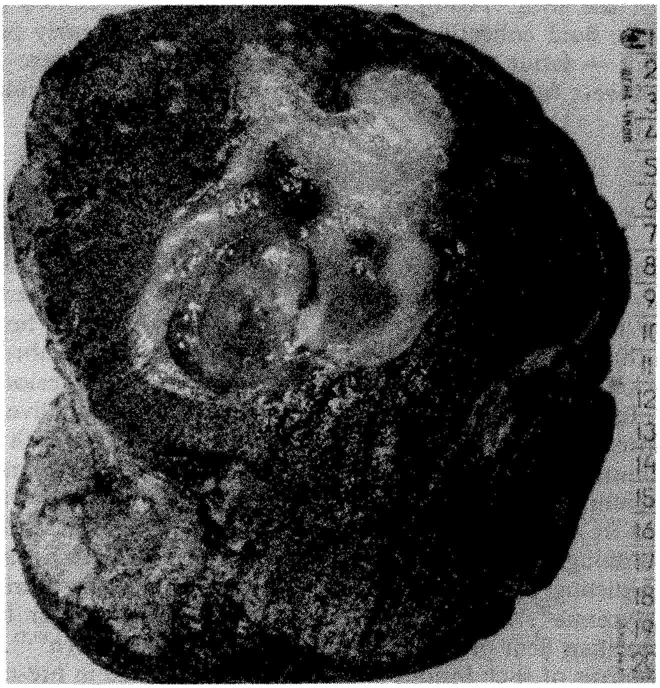

**Table 3** Details of the lesion and operative procedure.

| *Case No.* | *Maximum dimension (cm)* | *Hepatic Segments* | *Embolization* | *Blood Loss (ml)* | *Operative procedure* |
|---|---|---|---|---|---|
| 1. | 18 | II-IV | + | 850 | Left lobectomy |
| 2. | 17 | II-IV | + | 700 | Left lobectomy |
| 3. | 21 | II-IV | + | 1500 | Left lobectomy |
| 4. | 17 | II-IV | + | 1500 | Left lobectomy |
| 5. | 15 | II, III | + | 1000 | Left lobectomy |
| 6. | 16 | II, III | + | 1100 | Left lobectomy |
| | 8 | IV, V | | | Resection of Seg V |
| 7. | 18 | II-VI | + | 2500 | Left lobectomy |
| | | | | | Resection of Seg V, VI |
| 8. | 15 | II, III | + | 500 | Lt. lateral segmentectomy |
| 9. | 15 | II, III | + | 1000 | Lt. lateral segmentectomy |
| 10. | 16 | II, III | + | 250 | Lt. lateral segmentectomy |
| 11. | 16 | IV-VI | + | 1000 | Resection of seg IV-VI |
| 12. | 15 | V, VI | + | 500 | Resection of seg V, VI |
| 13. | 15 | V-VIII | + | 2500 | Rt. trisegmentectomy |
| | 5 | IV | | | |
| 14. | 20 | V-VIII | – | 2000 | Right lobectomy |
| 15. | 30 | IV-VIII | + | 18000 | Rt. trisegmentectomy |
| 16. | 31 | V-VIII | – | 11000 | Right lobectomy |

hemangioma of the liver. Postoperative complications developed in 4 (25%)– subdiaphragmatic fluid collection in 2, peritonitis in 1 and early post-operative hemorrhage in 1 — latter two required relaparotomy. There were no operative deaths, 14 cases were followed from 6–45 months (mean 18) and remain symptom free.

### DISCUSSION

Cavernous hemangioma is the most common benign tumor of the liver occurring more frequently in females in the fourth decade of life[11,12]. Age and sex of our study match with that reported in the literature. The clinical features of hepatic hemangioma are not characteristic of liver tumors[7]. Lesions measuring more than 4cm in diameter are known as giant hemangiomas because such lesions are often symptomatic[13]. In a series of 16 cases of cavernous hemangioma of liver reported by Schwartz and Husser,[7] the mean size of operated lesion was 10cm with 50% of them palpable on clinical examination. Iwatsuki *et al*[12] reported 109 operated cases of hepatic hemangiomas with mean size of 12cm and abdominal pain was a common symptom noted in 45% of cases. Hematological, biochemical and coagulation abnormalities although rare, are reported with these lesions– the Kasabach-Merritt syndrome[14], consisting of anemia, thrombocytopenia, primary fibrinolysis[12] and reactive hypoglycemia[10]. On ultrasonographic examination larger hemangiomas measuring more than 8cm produce an heterogenic echo pattern[10,15]. CT is more accurate in diagnosis of such lesions showing low dense area in pre-contrast scans and characteristic enhancement from periphery to centre of hemangioma on post-contrast dynamic scanning[15,16]. MRI is the latest contribution to liver imaging having an accuracy of nearly 90% in diagnosis of hepatic hemangiomas[17]. T2 weighed images by MRI are characteristic and help to differentiate hemangiomas of liver from other hypervascular tumors like hepatocellular carcinoma[18]. Hepatic angiography has a diagnostic accuracy of 100%[19] but, since it is invasive, it is being replaced by noninvasive and equally informative multi-modal imaging techniques– US, CT and MRI[5–10]. Although severe bleeding has occurred following needle aspiration biopsy,[6,8,20] successful diagnostic use of FNAC has been reported in 15 cases of cavernous hemangioma of the liver without any complication[21]. None of our 11 cases experienced any complication following FNAC but its diagnostic yield was 64%. Following therapeutic hepatic artery embolization for benign hepatic tumors including hemangiomas, complications like tumor liquitication, abscess formation and emboli migration are reported[9,10]. Two of our cases developed hypofibrinogenemia following embolization and one (case 16) had thrombocytopenia, hypofibrinogenemia and a low prothrombin index after angiography. We believe that embolization, if carried out alone is not the effective method of treatment of large symptomatic cavernous hemangiomas, as the disease *per se* is not eradicated and the chances of tumor necrosis and abscess formation are high.

Surgery is the treatment of choice for giant cavernous hemangiomas[7,12–14,20] because the majority of such lesions are symptomatic. Thirteen of 16 (81%) operated giant cavernous hemangiomas reported by Schwartz and Husser[7] had pain and/or mass. Similarly 18 of 22 (82%) operated cases reported by Adam *et al.*[13] were noted to have various abdominal symptoms including spontaneous rupture. Although the potential for rupture of these lesions is minimal– 1 to 3%[15,23], the rupture either spontaneous or induced by trivial trauma is associated even in the present day with high mortality[13,22,24]. Rapid increase in their size has been reported[7,13]. Cavernous hemangiomas pose special problems during liver resection because of their vascular nature. In Starzl's series of 15 operated cases[20], the average blood loss was 1700ml and Schwartz in his 16 resected cases of hepatic hemangiomas noted on average blood loss of 1750 ml[7]. Of our 14 cases with preoperative selective hepatic artery embolization, 13 major hepatic resections were associated with an average blood loss of 1146 ml. Although our study lacks a control group without embolization, our case material is comparable with that of previous authors[7,20]. Size of operated lesion is larger in our cases (mean 19cm versus 10cm) and operative blood loss is less (mean 1146 ml versus 1750 ml). But in two cases including one with embolization (case 15 and 16), profuse retrograde venous bleeding occurred during right lobectomy in one and right trisegmentectomy in the other, probably due to the presence of arteriovenous communications within the lesion. In each of these cases huge right lobe hemangioma added technical difficulty to gain access to the right hepatic vein. Starzl *et al.*[25] reported a similar case of giant hepatic hemangioma with arteriovenous malformation which led to massive blood loss of 20,000 ml during left trisegmentectomy with successful outcome. In our two cases massive intraoperative hemorrhage was replaced by autotransfusion of blood from operating field with the help of Cell-saver and fresh donor blood and plasma transfusion. Routine use of Cell-saver is described by Schwartz[7] during hepatic resections for benign lesions including hemangiomas.

In conclusion, giant symptomatic hepatic hemangiomas, unlike other benign tumors of liver pose problems of increased bleeding during hepatic resection because of their vascular nature and they are better operated on in specialized surgical centres that routinely perform hepatic surgery. Preoperative selective hepatic artery embolization helps to decrease intraoperative bleeding in the majority. However, in cases of severe bleeding presence of adequate blood reserve and use of Cell-saver can help tide over the crisis.

### *References*

1. Frerichs, F.T. (1861) A clinical treatise on disease of liver, Vol. 2. Translated by C. Murchison. London: New Sydenham Society.
2. Ochsner, J.L. and Halpert, B. (1958) Cavernous hemangioma of liver. *Surgery* **43**, 577–582.
3. Ishak, K.G. and Robin, L. (1975) Benign tumors of liver. *Med. Clin. North Am.* **59**, 995–1013.
4. Itai, Y., Ohtomo, K. and Furui, S. *et al.* (1985) Non invasive diagnosis of small cavernous hemangiomas of liver. Advantage of MRI. *Am. J. Roentgenol* **145**, 1195–1199.
5. Ekelund, L., Heitala, S.O. and Athlin, L. (1987) Multimodality imaging of large cavernous hemangioma of liver. *J. Med. Imag.* **1**, 348–353.
6. Trastek, V.F., van Heerden, J.A., Sheedy, P.F. III. and Adson, M.A. (1983) Cavernous hemangioma of liver: resect or observe? *Am. J. Surg.* **145**, 49–53.
7. Schwartz, S.I. and Husser, W.C. (1987) Cavernous hemangioma of liver: A single institute report of 16 resections. *Ann. Surg.* **205**, 456–465.
8. Iwatsuki, S. and Starzl, T.E. (1988) Personal experience with 411 hepatic resections. *Ann. Surg.* **208**, 421–434.
9. Allison, D.J., Jordan, H. and Hennessy, O. (1985) Therapeutic embolization of hepatic artery: a review of 75 procedures. *Lancet* **1**, 595–599.
10. Reading, N.G., Forbes, A., Nunerley, H.B. and Williakns, R. (1988) Hepatic Hemangioma: A critical review of diagnosis and management. *Q. J. Medicine* **67**, 431–445.
11. Shumacker, H.B. Jr. (1942) Hemangioma of liver. *Surgery* **11**, 209–222.
12. Iwatsuki, S., Sheahan, D.G. and Starzl, T.E. (1989) The changing face of hepatic resection. *Cur. Prob. Surg.* **26**, 5.

13. Adam, Y.G., Huvos, A.G. and Fortner, J.G. (1970) Giant cavernous hemangioma of liver. *Ann. Surg.* **172**, 239–243.
14. Kawarada, Y. and Mizumoto, R. (1984) Surgical treatment of giant hemangioma of liver. *Am. J. Surg.* **148**, 287–291.
15. Montorsi, M., Fumagalli, U., Spiropoulos, I. and Bona, S. Cavernous liver hemangioma. XXVI World Congress of International College of Surgeons. Milan 1988. Lecture book II: 833–834.
16. Itai, Y., Ohtoma, K. and Akari, T. (1983) CT and Sonography of cavernous hemangioma liver. *AJR* **141**, 315–320.
17. Stark, D.D., Felder, R.C., and Wittenberg, J. (1985) MRI of cavernous hemangioma of the liver: tissue specific characterization. *AJR* **145**, 213–222.
18. Ohtomo, K., Itai, Y. and Yoshida, H. *et al.* (1989) MRI differentiation of HCC from cavernous hemangioma. FLASH and T2 values. *AJR* **152**, 505–507.
19. Johnson, C.M., Sheedy, P.F. and Stanson, A.W. *et al.* (1981) Computed tomography and arteriography of cavernous hemangioma of liver. *Radiology* **138**, 115–121.
20. Starzl, T.E., Koep, L.G. and Weil, R. III *et al.* (1980) Excisional treatment of cavernous hemangioma of liver. *Ann. Surg.* **192**, 25–27.
21. Cronan, J.J., Esparza, A.R. and Dorfman, G.S. *et al.* (1988) Cavernous hemangioma of the liver: role of percutaneous biopsy. *Radiology* 135–138.
22. Sewell, J.H. and Weiss, K. (1961) Spontaneous rupture of hemangioma of the liver. A review of literature and presentation of illustrative case. *Arch. Surg.* **83**, 729–733.
23. Huguet, C. and Mouiel, J. (1983) Les tumeurs primitives du foie chez l'adulte. Paris, Masson.
24. Andersson, R., Tranberg, K. and Bengmark, S. (1988) Hemoperitoneum after spontaneous rupture of liver tumor: results of surgical treatment. *HPB Surgery* **1**, 81–83.
25. Starzl, T.E., Iwatsuki, S. and Shaw, B.W. Jr. *et al.* (1982) Left hepatic trisegmentectomy. *Surg. Gynaecol. Obstet.* **155**, 21–27.

*(Accepted by S. Bengmark 5 November 1990)*

### INVITED COMMENTARY

The authors are to be congratulated for the zero operative mortality achieved in these 16 hepatic resections done for giant cavernous hemangiomas (all measured 15 cm or more in diameter). This study supports the current contention that major hepatic resections should today be performed with an acceptably low mortality and morbidity.

The authors suggest a multimodality diagnostic approach when hepatic hemangiomata are suspected. This approach includes a variety of invasive (angiography) and noninvasive (ultrasonography) radiological techniques as well as fine needle aspiration biopsy. The latter is certainly controversial—despite the lack of complications following FNA in this series, wide application should be cautioned. The authors do not suggest which investigation they prefer or in which sequence. In this study, the sensitivity for computerized tomography was 82%. Although they state that all patients in their study were symptomatic—the manuscript suggests that these symptoms were minimal in nature which would be in keeping with most reported series.

Of particular interest is the suggestion that routine preoperative selective hepatic artery embolization should be performed since it might decrease operative blood loss. This is based on the nonrandomized comparison of 14 patients who underwent preoperative embolization, to two patients who did not. As the authors themselves are quick to point out, such a comparison is not valid. It is hoped that they might consider randomization of their patients in the future, thus providing us with extremely valuable information. It has been our experience that blood loss during hepatic resections for cavernous hemangiomas is often minimal and that the "bad reputation" that these fascinating vascular tumors have is probably unjustified.

J.A. van Heerden
Mayo Clinic
Rochester Ma
USA

